# Acute Reversible Convergent Strabismus Following Accidental Lorazepam Ingestion in a Child: A Rare Ocular Manifestation of Benzodiazepine Toxicity

**DOI:** 10.7759/cureus.105405

**Published:** 2026-03-17

**Authors:** Omayma El Athmani, Leila Debono, Youssef Jeddi, Nour Mekaoui, Lamya Karboubi

**Affiliations:** 1 Pediatrics, Children’s Hospital, University Hospital Center Ibn Sina, Faculty of Medicine and Pharmacy, Mohammed V University, Rabat, MAR; 2 Pediatric Emergency Medicine, Children’s Hospital, University Hospital Center Ibn Sina, Faculty of Medicine and Pharmacy, Mohammed V University, Rabat, MAR

**Keywords:** acute strabismus, benzodiazepine intoxication, esotropia, lorazepam poisoning, pediatric poisoning, toxicology

## Abstract

Benzodiazepine intoxication in children commonly manifests with central nervous system depression, hypotonia, and ataxia. Ocular motor abnormalities are uncommon and may mimic serious neurological disorders, frequently prompting urgent neuroimaging. We report the case of an eight-year-old boy who presented with sudden-onset convergent strabismus approximately 30 minutes after accidental ingestion of 10 mg of lorazepam. On admission, he was hemodynamically stable with a Glasgow Coma Scale score of 15/15. Neurological examination was unremarkable except for acute esotropia. Brain computed tomography revealed no abnormalities, and urine toxicology screening was positive for benzodiazepines. The patient was managed conservatively with close clinical observation without administration of flumazenil. Complete spontaneous resolution of the ocular deviation occurred within 12 hours. This case highlights that isolated acute strabismus may represent a rare but reversible manifestation of benzodiazepine intoxication in children. Awareness of this presentation may help clinicians consider toxicological causes in the differential diagnosis and avoid unnecessary invasive investigations.

## Introduction

Benzodiazepines are widely prescribed medications with anxiolytic, sedative, anticonvulsant, and muscle-relaxant properties and are frequently implicated in accidental pediatric poisonings because of their widespread domestic availability [1]. In children, benzodiazepine intoxication typically manifests with central nervous system depression, drowsiness, hypotonia, and impaired coordination [2].

Visual disturbances such as blurred vision, diplopia, and nystagmus have occasionally been reported during benzodiazepine exposure, although ocular motor abnormalities remain relatively uncommon in pediatric patients [1]. Ocular manifestations related to benzodiazepine exposure are rarely described and may mimic serious neurological disorders, often prompting urgent neuroimaging.

Acute acquired strabismus in children is generally considered an alarming clinical sign that may indicate intracranial pathology, including tumors, increased intracranial pressure, or cranial nerve palsy. However, toxicological etiologies should also be considered in the differential diagnosis. Recognizing atypical manifestations of drug intoxication may help avoid unnecessary diagnostic investigations and facilitate appropriate management.

We describe a rare case of benzodiazepine intoxication presenting with acute isolated convergent strabismus in a child.

## Case presentation

An eight-year-old previously healthy boy (weight: 25 kg) was brought to the pediatric emergency department after his parents noticed a sudden inward deviation of both eyes. The symptoms developed approximately 30 minutes after accidental ingestion of a 10 mg tablet of lorazepam at home.

On admission, the patient was alert and cooperative with a Glasgow Coma Scale score of 15/15. Vital signs were within normal limits: blood pressure 105/65 mmHg, heart rate 88 beats per minute, respiratory rate 18 breaths per minute, and oxygen saturation 99% on room air.

Neurological examination revealed acute convergent strabismus in primary gaze without ptosis, ophthalmoplegia, or nystagmus. The child did not report diplopia. Extraocular movements were full in all directions of gaze, including bilateral abduction. A bedside cover-uncover test confirmed the presence of convergent strabismus without evidence of cranial nerve palsy. Muscle tone, strength, coordination, and gait were preserved, and no ataxia was observed.

Based on the reported ingestion of a 10 mg tablet in a 25 kg child, the estimated exposure corresponded to approximately 0.4 mg/kg. The parents reported ingestion after noticing a missing tablet shortly before symptom onset; however, the exact dose absorbed could not be definitively confirmed.

Given the abrupt onset of ocular deviation, brain computed tomography was performed to exclude intracranial pathology and showed no abnormalities. Urine toxicology screening consisted of a qualitative benzodiazepine immunoassay. The test confirmed benzodiazepine exposure but was not specific for lorazepam and did not provide quantitative drug levels.

The patient was admitted to the pediatric observation unit for close monitoring. Considering his stable clinical status and preserved level of consciousness, supportive management without administration of flumazenil was chosen. During observation, progressive clinical improvement was noted.

**Figure 1:**
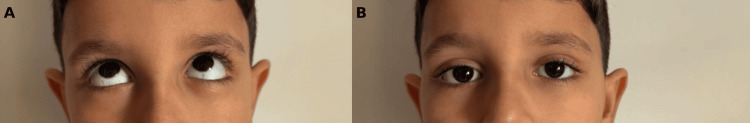
Clinical evolution of ocular alignment (A) Acute convergent strabismus observed at presentation after accidental lorazepam ingestion. (B) Normal ocular alignment observed 12 hours after clinical observation, demonstrating complete resolution of the strabismus.

The evolution of ocular alignment during the observation period is illustrated in Figure 1. Acute convergent strabismus was evident at presentation (Figure 1A). Complete resolution with normal ocular alignment occurred within approximately 12 hours of observation (Figure 1B).

The patient remained clinically stable and was discharged home. At the follow-up examination one week later, the child remained asymptomatic and had normal ocular alignment.

## Discussion

Benzodiazepines exert their pharmacological effects by enhancing gamma-aminobutyric acid (GABA) activity at the GABA-A receptor, thereby increasing inhibitory neurotransmission within the central nervous system [1]. This mechanism explains the typical manifestations of benzodiazepine intoxication, including drowsiness, hypotonia, impaired coordination, and varying degrees of central nervous system depression [2]. In pediatric patients, most accidental exposures are mild and resolve with supportive care alone.

Although visual disturbances such as blurred vision, diplopia, and nystagmus have occasionally been described during benzodiazepine intoxication, isolated acute strabismus remains extremely rare in the pediatric population. The exact pathophysiological mechanism remains uncertain. However, excessive GABAergic inhibition within brainstem ocular motor pathways, including the paramedian pontine reticular formation and the abducens nucleus, may transiently disrupt the balance between excitatory and inhibitory signals responsible for binocular coordination. This imbalance could lead to temporary dysfunction of the extraocular muscles and result in transient ocular misalignment.

Acute acquired strabismus in children is often considered a warning sign of potentially serious neurological conditions. Differential diagnoses include acute acquired comitant esotropia, convergence spasm, sixth cranial nerve palsy, intracranial hypertension, posterior fossa tumors, demyelinating disorders, and neuromuscular junction diseases such as myasthenia gravis. Consequently, clinicians frequently perform urgent neuroimaging to exclude structural brain abnormalities.

In the present case, several clinical features strongly supported a toxic and reversible etiology. First, the onset of symptoms occurred shortly after the reported lorazepam ingestion. Second, the patient remained fully conscious with an otherwise normal neurological examination. Third, neuroimaging revealed no structural abnormalities. Finally, the rapid and complete resolution of symptoms within 12 hours suggested a transient pharmacological effect rather than an underlying neurological disease.

The estimated ingested dose of approximately 0.4 mg/kg exceeds typical therapeutic dosing in pediatric patients. Interestingly, despite this potentially high exposure, the child maintained normal consciousness and did not develop significant central nervous system depression. This unusual clinical course may reflect incomplete ingestion of the tablet or interindividual variability in benzodiazepine sensitivity.

Flumazenil, a competitive benzodiazepine receptor antagonist, may reverse sedation. Still, its use remains controversial because of the potential risk of inducing seizures, particularly in cases of mixed overdoses or chronic benzodiazepine exposure [3,4]. Management of benzodiazepine intoxication in children is primarily supportive and includes careful monitoring of respiratory and neurological status until spontaneous recovery occurs [5]. In this patient, flumazenil was not administered because the child maintained normal mental status and stable respiratory function, making the risk-benefit ratio of antidote administration unfavorable.

Recognition of atypical manifestations of drug intoxication is important in pediatric emergency medicine. Awareness that benzodiazepine exposure may rarely present with isolated ocular motor disturbances could help clinicians broaden the differential diagnosis of acute strabismus and potentially avoid unnecessary invasive investigations.

## Conclusions

Accidental lorazepam ingestion in children may rarely present with isolated acute convergent strabismus despite preserved consciousness and an otherwise normal neurological examination. This case suggests that acute strabismus may represent an uncommon but reversible manifestation of benzodiazepine intoxication. Awareness of this unusual presentation may help clinicians consider toxicological causes in the differential diagnosis and potentially avoid unnecessary diagnostic procedures.
